# Occupational Exposure of Hairdressers to Airborne Hazardous Chemicals: A Scoping Review

**DOI:** 10.3390/ijerph19074176

**Published:** 2022-03-31

**Authors:** Sanja Kezic, Roberto Nunez, Željka Babić, Sarah Hallmann, Martin S. Havmose, Jeanne D. Johansen, Swen M. John, Marija Macan, Cara Symanzik, Wolfgang Uter, Patricia Weinert, Rajka Turk, Jelena Macan, Henk F. van der Molen

**Affiliations:** 1 Amsterdam UMC, Department of Public and Occupational Health, Coronel Institute of Occupational Health, Amsterdam Public Health Research Institute, University of Amsterdam, 1105 AZ Amsterdam, The Netherlands; robertonnz@gmail.com (R.N.); h.f.vandermolen@amsterdamumc.nl (H.F.v.d.M.); 2 Institute for Medical Research and Occupational Health, HR 10001 Zagreb, Croatia; zbabic@imi.hr (Ž.B.); mmacan@imi.hr (M.M.); rturk@imi.hr (R.T.); jmacan@imi.hr (J.M.); 3 Department of Medical Informatics, Biometry and Epidemiology, University of Erlangen, 91054 Erlangen, Germany; sarah.hallmann@fau.de (S.H.); wolfgang.uter@fau.de (W.U.); 4 National Allergy Research Centre, Department of Skin and Allergy, University of Copenhagen, Gentofte Hospital, 2900 Copenhagen, Denmark; martin.stibius.havmose@regionh.dk (M.S.H.); jeanne.duus.johansen@regionh.dk (J.D.J.); 5 Department of Dermatology, Environmental Medicine and Health Theory, Osnabrück University, 49076 Osnabrück, Germany; s.m.john@uni-osnabrueck.de (S.M.J.); csymanzik@uni-osnabrueck.de (C.S.); 6 Institute for Interdisciplinary Dermatological Prevention and Rehabilitation (iDerm), Osnabrück University, 10777 Berlin, Germany; pweinert@uos.de

**Keywords:** airborne exposure, indoor exposure, health effects, inhalation, hairdressers, hair salons

## Abstract

Introduction: Exposure to hazardous chemicals released during hairdressing activities from hair care products puts hairdressers at risk of adverse health effects. Safety assessments of hair products are mainly focused on consumers, but exposure for professional hairdressers might be substantially higher. Objective: To identify and assess available research data on inhalation exposures of professional hairdressers. Methods: A systematic search of studies between 1 January 2000 and 30 April 2021 was performed in Medline, Embase, Web of Science and in Cochrane registry, toxicological dossiers of the Scientific Committee on Consumer Safety (SCCS) of the European Commission as well as the German MAK Commission. Studies reporting quantitative data on airborne concentrations of chemicals in the hairdresser’s workplace were considered. The outcome was an airborne concentration of chemicals in the working environment, which was compared, when possible, with current occupational exposure limits (OEL) or guidance levels. Results: In total, 23 studies performed in 14 countries were included. The average number of hairdressing salons per study was 22 (range 1–62). Chemicals most frequently measured were formaldehyde (*n* = 8), ammonia (*n* = 5), total volatile organic compounds (TVOC) (*n* = 5), and toluene (*n* = 4). More than fifty other chemicals were measured in one to three studies, including various aromatic and aliphatic organic solvents, hydrogen peroxide, persulfate, and particulate matter. Most studies reported environmental air concentrations, while personal exposure was measured only in seven studies. The measured air concentrations of formaldehyde, ammonia, and TVOC exceeded OEL or guidance values in some studies. There was large variability in measuring conditions and reported air concentrations differed strongly within and between studies. Conclusion: Hairdressers are exposed to a wide spectrum of hazardous chemicals, often simultaneously. Airborne concentrations of pollutants depend on salon characteristics such as ventilation and the number of customers but also on used products that are often country- or client-specific. For exposure to formaldehyde, ammonia, and TVOC exceeding OELs or guidance values for indoor air was observed. Therefore, occupational exposure should be taken into account by safety regulations for hair care products.

## 1. Introduction

The hairdressing sector with as many as 400,000 salons in the European Union (EU) employed 1.7 million workers in 2019, representing 0.9% of total employment [1]. Hairdressers come into contact with hair care products on a daily basis and are exposed to a wide variety of potentially hazardous chemicals through inhalation and/or skin contact. Some hair-smoothing or straightening products release formaldehyde which can cause irritation to the skin, eyes, and respiratory system. Formaldehyde is, in addition, a strong dermal sensitizer and is regarded as a human carcinogen [2]. Another commonly used hazardous chemical is ammonia, which is released during bleaching, oxidative dyeing, or perm procedures, and can lead to irritation of the skin, eyes, and respiratory system, as well as sensitization after dermal contact or by inhalation [3]. Hair bleaches also contain persulfate salts known to cause irritation to the skin, eyes, and respiratory system, and also sensitization after dermal contact or by inhalation, and are potent asthmagens [3]. Other chemicals often present in hairdressing salons include ethanol, volatile organic chemicals (VOCs) such as aromatics, esters, ketones, and terpenes, and contact allergens such as limonene [4,5,6,7,8].

As compared to the general population, hairdressers have a higher incidence of various diseases, including rhinitis and asthma [6,9,10] and contact dermatitis [11,12]. Several studies report moreover reproductive effects [13,14,15], which are of importance as hairdressers are predominantly women of childbearing age [16].

Intrinsic toxicological properties of chemicals are not the only determining factor for the development of adverse health effects, as the risk for adverse health effects depends on the pattern and the degree of exposure. Safety assessment of hair products in the EU follows the guidelines of the Scientific Committee on Consumer Safety (SCCS) for the regulation of cosmetic products [17], which are primarily focused on the consumer, even for products that are used by both professionals and non-professionals. However, the degree of exposure for a hairdresser can be substantially higher than that for a consumer, [18] thereby increasing the risk for adverse health effects. Hence, data on exposure are indispensable for the health risk assessment. For this scoping review, we collected literature data on dermal and inhalation exposure of hairdressers at the workplace. This paper is part of a series of reviews focused on the exposure and health risks in hairdressers, of which the main protocol has been published elsewhere [19]. Dermal exposure will be the subject of another review from this series [20]. This scoping review focused on inhalation exposure, i.e., the air concentrations of hazardous chemicals in the working environment. Data on airborne concentrations will be compared with existing occupational exposure limit values.

## 2. Methods

A scoping review was performed using the Arksey and O’Malley framework, which in contrast to systematic reviews, does not perform a quality assessment of the included studies but provides an overview of the literature [21]. The research question is defined in a way that results from studies of various designs are included and charted in tables or figures. We also used the checklist of Preferred Reporting Items for Systematic Reviews—extension for Scoping Reviews (PRISMA-ScR) [22]. A protocol was published before beginning this scoping review amongst another set of reviews about exposure and health risks in hairdressers [19].

### 2.1. Information Sources and Search

In collaboration with a clinical librarian, a systematic search of the peer-reviewed literature published in English or Dutch between 1 January 2000 and 30 April 2021 was performed using the following electronic databases: Medline, EMBASE, and Web of Science-Core Collection. Additionally, the Cochrane registry and the Toxicological dossiers of the Scientific Committee on Consumer Safety (SCCS) of the European Commission, as well as the German MAK Commission, have been searched [23]. We also performed a manual search through Google Scholar to identify any potentially relevant articles that may have been missed in the first systematic search. The detailed search syntax is shown in the Supplementary Table S1.

The population of interest was professional hairdressers. For exposure, we considered chemicals that can be released from hair products used by hairdressers in hairdressing and beauty salons. The outcome is airborne concentration of chemicals. Biomonitoring studies and studies with experimental design were excluded.

### 2.2. Screening of Articles

The retrieved articles were independently screened for inclusion by two authors (RN and SK) based on abstracts, titles, and keywords. For screening of title and abstracts, we used the web-based literature screening tool Rayan [24]. Depending on the routes of exposure investigated, the articles were tagged with either the “DER” or “INH” keyword for dermal and inhalational exposure. For this review, only “INH” articles were considered. Articles were approved by both authors based on inclusion criteria. In the case of conflicts, the two authors discussed the articles until they settled on an agreement for approval/disapproval.

### 2.3. Eligibility of Studies

**Figure 1:**
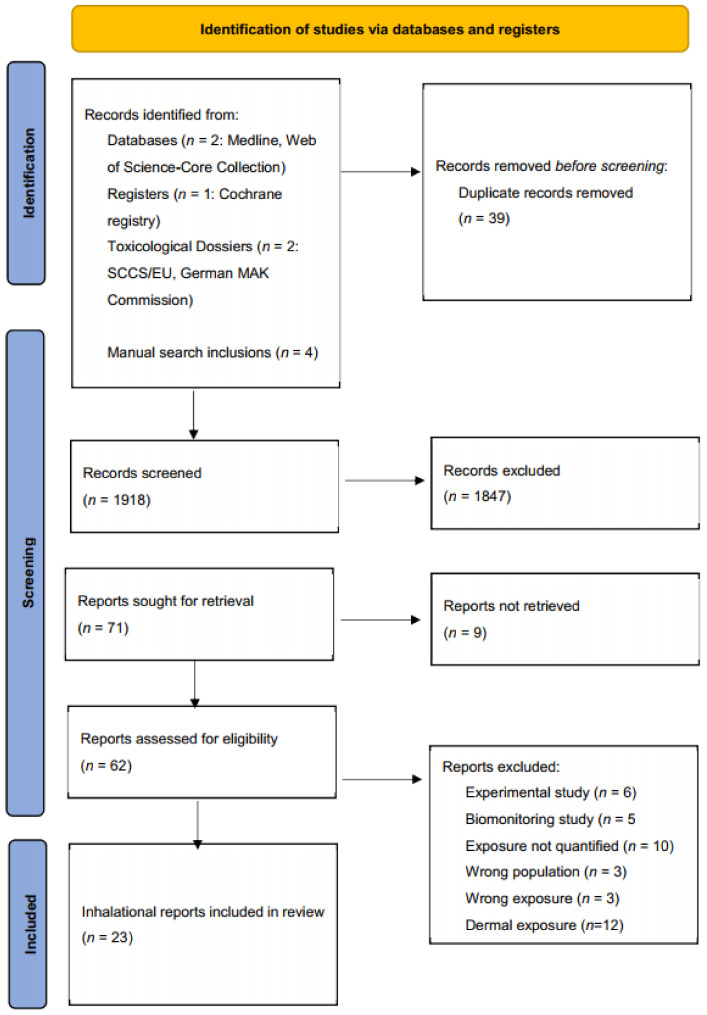
PRISMA Flow diagram for the screening and selection of articles.

Two authors (SK and RN) documented reasons for exclusion and inclusion of full-text articles (Figure 1).

### 2.4. Data Extraction

Data from selected articles were extracted by the two authors (SK and RN), and included year of publication, country of origin, study design, methods, studied chemical, study setting and involved population, information on basic characteristics of population and study location, number of participants, and outcomes. Outcomes were extracted when appropriate in subcategories regarding the type of measurements, i.e., environmental or personal sampling.

Data on air concentrations in parts per million (ppm) used in some studies were converted to mg/m^3^ as follows [25]:

Concentration (mg/m^3^) = (ppm) × (molecular weight) × (1/24.45*) (1)

* Molar volume of gas at 1 atmosphere and 25 °C.

Data on airborne concentration found in included studies were compared with current occupational exposure limits (OEL) or guidance values for occupational settings in the EU (Supplementary Table S2) [26]. Additionally, in Table S2, we present often used occupational permissible exposure limits (PEL) of the Occupational Safety and Health Administration (OSHA) in the USA [27]. Two types of OEL were considered: short-time exposure limit (STEL) based on 15 min average concentrations and a time-weighted average concentration over 8 h (TWA). For TVOC, there was no OEL, and we used the target and guideline values for indoor air [28]. Next, for ethanol which also does not have an OEL at the EU level, we used the OEL applied in the Netherlands [29].

## 3. Results

### 3.1. Selected Studies

An overview of the screening and selection processes is shown in the PRISMA flow chart (Figure 1). After removal of duplicates, 1918 unique articles remained, of which 71 were sought for full-text retrieval after the screening for title and abstract. Eventually, 23 articles covering inhalation exposure were included in this scoping review. Studies were performed in fifteen countries, of which five originate from the EU.

Data on air concentrations measured in the working environment are collated in Table 1, Table 2 and Table 3. Out of the 23 studies, eight reported air concentrations of formaldehyde, five reported ammonia, five reported total volatile organic compounds (TVOC), four reported toluene, and three reported xylene (isomers), acetone, benzene, ethanol, isopropanol, ethyl acetate, and ethyl benzene. In addition, exposure to various chemicals (*n* = 45) was investigated in only one or two studies which are listed in Supplementary Table S3.

In most of the studies (19 out of 23), environmental (or static) sampling was applied where the measuring instrument was placed at a location of interest within the salon. Environmental monitoring does not provide information on personal exposure but is primarily used to identify problems and priorities in the workplace. In order to evaluate the risk of exposure of the individual worker, measurements have to be performed in the breathing zone of the worker (personal monitoring), and the TWA concentrations have to be compared with the OEL. As personal sampling was carried out in only eight studies, we also related TWA concentrations obtained by environmental sampling to OELs.

Duration of measurements ranged from 15 min to 8 h, and all reported concentrations were expressed as TWA over the measurement period. In general, short measurement times were carried out to gather information about exposure during specific hairdressing activities.

**Figure 2:**
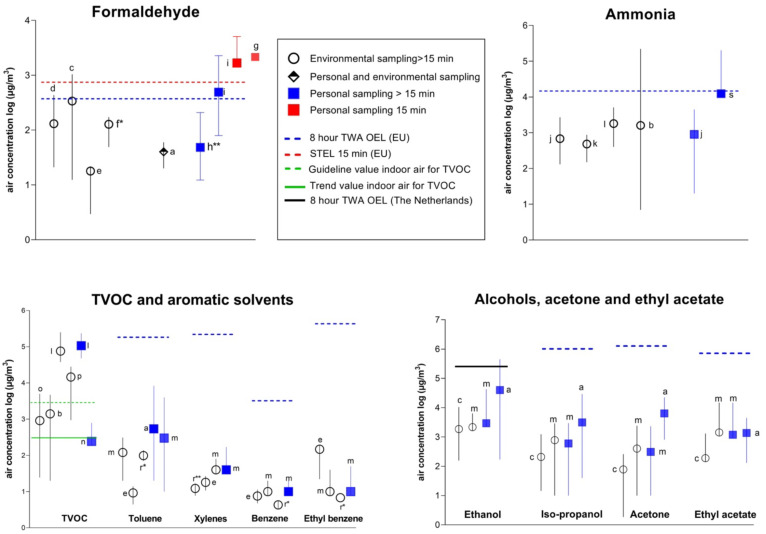
Air concentrations of formaldehyde, ammonia, TVOC, aromatic and aliphatic solvents obtained from personal and environmental samples. The letters by the symbols refer to reference numbers as follows: a [5]; b [6]; c [30]; d [31]; e [32]; f [33]; g [34]; h [35]; I [36]; j [37]; s [38]; k [39]; l [40]; m [41]; n [42]; o [43]; p [44]; r [45]. Data are presented as mean and ranges unless indicated otherwise. * Median and range. ** Mean of medians and range. OEL: occupational exposure limit.

In Figure 2, data on airborne concentrations measured in different studies are shown for formaldehyde, ammonia, TVOC, aromatic, and aliphatic organic solvents. These (group of) chemicals were reported in at least three studies. The air concentrations obtained by environmental and personal sampling are shown separately. In the figures, corresponding OEL values (8 h TWA and 15 min STEL) are given. In the case of TVOC, the target and guideline values for indoor air were used [28], and for ethanol, the 8 h TWA applied in the Netherlands [29]. The air concentrations in Figure 2 are presented on a logarithmic scale, while in Table 1, Table 2 and Table 3 the absolute values are provided.

### 3.2. Investigated Chemicals

#### 3.2.1. Formaldehyde

Formaldehyde air concentrations were measured in eight studies which included 170 hairdressing or beauty salons from six countries, namely Ghana, Brazil, Canada, Egypt, Iran, and Taiwan.

##### Exposure and Measurements Conditions

Data on formaldehyde air concentrations and study characteristics are shown in Table 1. Studies showed varying measurement location, performed tasks, number of workers, and clients in the salon. Chang et al. [30] carried out measurements at different places in hair salons reporting the highest concentrations in the working area (511 μg/m^3^) followed by washing, technical and reception area (respectively 212, 136, and 147 μg/m^3^). In addition, formaldehyde concentrations were significantly affected by the number of workers, number of perming treatments, and floor surface areas. Asare-Donkor et al. [31] reported that the number of customers that visit the salon in a week, number of salon services offered, and age of the salon significantly correlated with the level of measured formaldehyde. Hadei et al. [32] reported that the number of hair styling treatments significantly affected formaldehyde air concentrations. Ventilation was reported in five of the eight studies, thereby displaying a mix between mechanical (fans or air purifiers) and natural ventilation (open windows or doors). The comparison between three types of ventilation showed that ventilations with air purifier, and with fan and open window, were more effective than just with the fan [32].

##### Reported Concentrations and Comparison with OEL’s

Data on formaldehyde air concentrations and comparison with OELs are summarised in Figure 2. Current STEL and 8 h TWA values for formaldehyde in the EU are 740 μg/m^3^ and 370 μg/m^3^ (Table S2). There was large variability in the reported air formaldehyde concentrations across studies which ranged from 0 to 5083.4 μg/m^3^. The environmental air concentrations (5 h TWA) of formaldehyde in the study of Chang et al. [30] exceeded the EU OELs in the working area (Table 1).

Hadei et al. [32] performed environmental sampling near the working area. The average formaldehyde concentration among 20 salons was 11.9 μg/m^3^. Formaldehyde concentrations were in all salons below the EU OELs.

In the study of Labreche et al. [5], the 8 h TWA concentrations were measured in 26 salons by applying personal and environmental sampling. The sampling took place during the busiest days of the week, namely from Thursday to Saturday, according to salon owners. The average formaldehyde concentration across all salons was 40.0 μg/m^3^_._ All measured air concentrations were below the OEL values for 8 h TWA in the EU.

Peteffi et al. [33] measured formaldehyde exposure in beauty salons during hair straightening based on environmental sampling. The median formaldehyde air concentration amounted to 127.5 μg/m^3^ and did not exceed the EU OELs in any of the investigated salons.

Aglan et al. [34] measured personal air concentrations during the hair straightening procedure (15 min). The average formaldehyde concentrations in the two groups of hairdressers were 2060.3 and 2244.3 μg/m^3^, which was higher than the EU STEL value.

In the study of Barbosa et al. [35], the median values of formaldehyde air concentrations measured in three subgroups of hairdressers during the hair straightening procedure ranged from 15.9 to 85.9 μg/m^3^. The measurements were performed by personal sampling. There was a strong positive correlation between the formaldehyde content in the hair straightening creams and the air formaldehyde concentrations.

Pexe et al. [36] performed personal measurements for 15 min during the hair straightening procedure, blow-drying and flat ironing stages with the expected greatest exposure. Next, personal sampling was conducted over an 8 h work shift. The average formaldehyde concentration during 15 min measurements was 1673 μg/m^3^ while 8 h TWA concentration was 492 μg/m^3^. The 15 min TWA exceeded in the majority of salons (16/23) the EU STEL value, while 8 h TWA was higher than 8 h OELs in 19 out of 23 salons.

#### 3.2.2. Ammonia

Air concentrations of ammonia were reported in five studies from five countries (Portugal, Spain, France, Palestine, Japan) which included 74 salons (Table 2) [6,37,38,39,40].

##### Exposure and Measurement Conditions

Various sampling locations inside salons were reported; the technical area where chemical mixtures are prepared was most often included (Table 2). The floor surface area in 74 reported salons ranged between 3 and 90 m^2^. One salon had only mechanical ventilation [39], while other salons had a mix of mechanical and natural ventilation [6,37]. In one study, measurements were carried out during the tasks with the highest expected exposure (hair dyeing, colouring, and bleaching) [38]. The highest air concentration of 202.1 mg/m^3^ was reported during colouring and bleaching. Measurements were performed over periods of time varying from 30 min to 8 h.

##### Reported Concentrations and Comparison with OEL’s

The current 8 h TWA OEL and STEL in the EU are 14 and 36 mg/m^3^, respectively (Table S1). Overall, average air ammonia concentrations within the salons ranged from 0.68 to 12.3 mg/m^3^. The highest recorded concentration was 220 mg/m^3^ (Table 2).

In the study of Mendes et al. [6], the environmental air samples were collected on Saturdays, when the hairdressers are usually busy, and a considerable amount of technical work takes place. Measurements were carried out in the technical areas and areas where hairdressers perform hair rinsing and drying. The average ammonia concentration was 1.6 mg/m^3^, and for 4% of workers, the prescribed 8 h OEL of the European Union of 14 mg/m^3^ was exceeded.

Nemer et al. [38] applied personal sampling carried out for 45–305 min. The median concentrations of ammonia in the salons ranged from 3 to 61 mg/m^3^. The ammonia concentrations above the EU 8 h TWA OEL (14 mg/m^3^) were found in six out of 13 salons. The highest recorded concentration of ammonia of 202.1 mg/m^3^ occurred during bleaching.

Oikawa et al. [39] performed environmental measurements of ammonia in one salon with five hairdressers. The average ammonia concentration amounted to 0.48 mg/m^3^, ranging from 0.15 to 0.87 mg/m^3^. Relatively higher concentrations were found during perm events and in samples collected near stations used for perm treatments. However, even the highest concentrations did not exceed the prescribed 8 h TWA OEL of the EU (14 mg/m^3^).

In the study of Ronda et al. [41], environmental measurements of ammonia were performed in 10 salons during one full week. The measurements lasted for 3 h and were performed before and after a lunch break. The ammonia concentrations were below the OEL, varying from 0.4 to 5.1 mg/m^3^.

#### 3.2.3. Total Volatile Organic Compounds (TVOC)

Five of the included 23 articles comprising 139 hairdressing salons from five countries (Italy, Portugal, Spain, UK, Taiwan) addressed TVOC exposure. Air concentrations and study characteristics are summarised in Table 3.

##### Exposure and Measurements Conditions

Duration of measurements ranged from 45 min to 48 h. Specific measurement conditions regarding exposure were reported in two studies. One of these studies performed measurements on both working and non-working days [42], while the other study placed samplers in three different areas (rinsing, drying, and technical areas) [6]. Ventilation was not reported in two of five studies. In one study, no ventilation was present, and two studies had air conditioning or mechanical ventilation.

##### Reported Concentrations and Comparison with OEL’s

For TVOC, there are no OELs, and measured air concentrations are used mainly as an indicator of indoor air quality. Recently, Tuomo et al. [28] proposed a TVOC target value of 300 µg/m^3^ and a guideline value of 3000 µg/m^3^ for the general indoor air in industrial workplaces. In Figure 2, these values were used for comparison. The average TVOC concentrations across the included studies ranged from 240 to 107,000 µg/m^3^. Four of five studies showed average air concentrations above the target value of 300 µg/m^3^, and two of five studies reported concentrations above the guideline value for the general indoor air in industrial workplaces of 3000 µg/m^3^.

De Gennaro et al. [43] reported average environmental concentrations of TVOC across twelve salons of 24.24 to 5002.86 μg/m^3^. The study has shown that air concentrations were influenced by work activities and the type of products used, but there was no correlation with the size of the salon or the number of customers.

In the study by Mendes et al. [6] carried out by environmental sampling, the average TVOC concentration across all salons was 1400 µg/m^3^. The measured concentrations ranged from 20 to 4700 µg/m^3^. The highest mean concentrations were measured in the technical area (1500 µg/m^3^) followed by the drying and rinsing areas (1400 and 1300 mg/m^3^).

Moda et al. [44] measured TVOC concentrations by environmental monitoring in five salons. The TWA concentrations across all salons ranged from 949 to 28,446 µg/m^3^, which was much higher than the target value. It has to be noted that in the investigated salons diverse range of activities were carried out, including pedicures, manicures, acrylic nail fixing, and hair perming.

Characteristics of the salon and measurement conditions in the study of Ronda et al. [41] are already described in the ammonia section. The mean concentrations of TVOC obtained from personal sampling amounted to 107,000 µg/m^3^ and 76,000 µg/m^3^ by environmental sampling. The environmental samples were taken at the mixing place, where high exposure was expected.

Ma et al. [42] measured TVOC concentration in hairdresser apprentices by personal monitoring during 12 working hours. The mean TVOC air concentration during working days was 308 µg/m^3^.

#### 3.2.4. Aromatic Solvents

Several studies investigated individual aromatic solvents, including toluene, xylene, benzene, and ethyl benzene. Toluene was investigated in the studies of Hadei et al. [32], Labreche et al. [5], Moradi et al. [45], and Ronda et al. [41], while the air concentrations of xylene, benzene, and ethyl benzene were measured in the respective studies of Hadei et al. [32] Moradi et al. [45] and Ronda et al. [41]. Characteristics of the salon and measurement conditions in these studies were already described in the previous section on formaldehyde, ammonia, and TVOC. As presented in Figure 2, measured air concentrations of all aromatic organic solvents were below the OELs.

#### 3.2.5. Aliphatic Organic Solvents

Among investigated aliphatic solvents were acetone, ethanol, isopropanol, and ethyl acetate [5,30,40]. In all studies, the measured concentrations of these solvents were below the OELs, with the exception of ethanol concentration measured in one salon in the study of Labreche et al. [5].

#### 3.2.6. Other Compounds

A large number of chemicals were investigated in only one or two studies and are listed in a Supplementary Table S3. Most of these chemicals were determined in the studies of Ronda et al. [29] (22 chemicals), Chang et al. [22] (9 chemicals), and Labreche et al. [5] (5 chemicals), of which the study characteristics were addressed already in previous sections.

Shao et al. [46] measured particulate matter (PM) in three salons primarily serving African/African American customers and three Dominican salons primarily serving a Latino clientele. The 8 h TWA concentrations for respirable particulate matter (RPM) defined as particles with a median aerodynamic diameter <4 μm was 299 μg/m^3^ (median value of all salons). The RPM air concentrations ranged from 18 to 383 μg/m^3^ in African/African American salons and from 9 to 2115 μg/m^3^ in Dominican salons. In European countries, the OELs for 8 h TWA exposure to respirable dust range from 300 to 6000 μg/m^3^ [26].

Albin et al. [47] investigated air concentrations of persulfates by personal and environmental measurements. The air concentrations obtained by personal sampling during mixing of bleaching powder with peroxide and application of the mixture ranged from 15 to 49 μg/m^3^ for sampling periods of 32–135 min. The air concentrations obtained by environmental sampling in the mixing area over 200–319 min ranged from 4 to 6.1 μg/m^3^.

Mounier-Geyssant et al. [37] measured exposure to hydrogen peroxide and persulfate in hairdressing apprentices. The average concentrations of hydrogen peroxide measured by personal and environmental sampling were respectively 0.05 and 0.04 mg/m^3^. For persulfate, the average air concentrations were 0.02 mg/m^3^ for both personal and environmental sampling. There was no significant association between the measured air concentrations and salon volumes or ventilation. Wan et al. determined the concentration of benzothiazoles and benzophenone-3 in barbershops/hair salons [47,48]. The median concentrations in bulk air (particulate and vapor phase) were 18.5 and 5.39 ng/m^3^, respectively. Liu et al. [49] measured the concentration of synthetic musks and methyl siloxanes in indoor dust [50].

## 4. Discussion

This scoping review included 23 articles that describe inhalation exposure to hazardous chemicals released from hairdressing products. More than 50 different chemicals were detected in the workplace air revealing the complexity of risk assessment in hair salons. In general, reported air concentration differed largely not only between but also within the investigated salons. This can partly be explained by different exposure conditions such as ventilation, number and type of hairdressing activities, length of product application, and density of hairdressers and clients in the salon [36]. Furthermore, hair care products vary between salons due to local safety regulations and the specific needs of the clients. Often, these factors were not reported in the studies, which hampers comparison of studies and identification of relevant factors which might impact air concentrations. In the present scoping review, we benchmarked the pertinent air concentrations against the pertinent occupational TWA exposure levels (OELs). Individual exposure assessment and comparison with OELs have to be based on data obtained by personal sampling, which were, however, carried out in only eight out of 23 included studies. Therefore, to provide an indication of the magnitude of exposure in this scoping review, we also related data obtained from environmental sampling to OELs. For uniformity, we used the current OELs in the EU, if available, as OELs may differ between countries and might not exist in some countries for certain chemicals.

### 4.1. Formaldehyde

The most often studied exposures concerned formaldehyde, which is present in most hair smoothers and straighteners. In Europe, formaldehyde is classified as a category 1B carcinogen and a category two mutagen [51]. Since 2019, formaldehyde is severely restricted in the EU (Commission Regulation 2019/831) [52], which might explain why none of the studies were performed in Europe. Mean air concentrations of formaldehyde in hairdressing and beauty salons showed large differences ranging from 11.9 to 2244.3 μg/m^3^. Formaldehyde air concentrations exceeded the OEL in the EU of 370 μg/m^3^ in two of seven investigated salons. Several studies addressed the exposure conditions which are relevant for the formaldehyde air concentrations. Barbosa et al. [35] reported an increase in formaldehyde concentrations with the number of straightening procedures. This is consistent with the findings of the study of Pexe et al. [36], showing a three times higher concentration of formaldehyde during straightening procedures as compared to TWA concentration over the whole shift.

### 4.2. Ammonia

Ammonia is released into the working atmosphere during permanent hair dyeing, wave preparation, and bleaching. Air concentrations of 0.3–10 mg/m^3^ in salon air can induce irritation in the airway mucosa during and after bleaching operations, and it has been shown that hairdressers have a higher risk of developing irritation of the upper airways and asthma [53,54,55]. The mean concentrations of ammonia reported in five studies were under the current OEL in the EU of 14 mg/m^3^, but in studies by Mendes et al. [6] and Nemer et al. [38], there were several salons where the measured concentrations largely exceeded this level. In a study by Nemer et al. [38], 6 out of the 13 salons were above the OEL. The authors explained the high concentrations in some salons by small size or having no or poor ventilation [38]. In addition, high concentrations were measured during certain tasks, in particular, colouring, cutting, bleaching, or spraying.

### 4.3. Total Volatile Organic Compounds (TVOC)

TVOC is commonly used in the assessment of indoor air quality, while a unified and worldwide accepted definition of TVOC is still missing. In practice, exposures often include much more hazardous compounds than those included in the measurement, and their composition and toxicity vary. The major potential health effects from VOC include acute and chronic respiratory effects, allergies, neurological toxicity, damage to the liver and kidney, reproductive effects, and carcinogenicity [56]. The air concentration of TVOC is widely proposed as a screening parameter, and the determination of individual components of TVOC is recommended [57]. Due to the ambiguity regarding the composition and levels of individual compounds, there are no OELs for TVOC. Exposure to TVOC between 300 and 3000 µg/m^3^ is associated with perceived discomfort as well as temporary symptoms of irritation in the eyes and the respiratory system [28,58,59]. For industrial facilities of constant work without respiratory protection, the guideline for long-term average TVOC was recommended to be below 3000 µg/m^3^, while a long-term TVOC target value of 300 µg/m^3^ has been recommended [28]. Two of the five included studies showed average TVOC concentrations above 3000 µg/m^3^. The highest TVOC air concentrations (38,000 to 250,000 µg/m^3^) were found in the study of Ronda et al. [41], who reported that none of the investigated salons had local exhaust ventilation. Clearly, hairdressers are exposed to the TVOC levels, which might lead to discomfort and adverse health effects.

### 4.4. Individual Organic Solvents

Several individual aromatic and aliphatic volatile organic solvents were studied. The group of aromatic solvents that were investigated included toluene, xylenes, benzene, and ethyl benzene, often referred to as BTEX solvents. Studies in humans show that BTEX exposure is associated with effects on immune, metabolic, respiratory, neurobehavioural, and endocrine functioning, as well as development [60]. Next, benzene is recognised as a human carcinogen [61]. Although the mean air concentrations of individual aromatic solvents were lower than the OEL, this should be interpreted with caution as hairdressers are often exposed simultaneously to different solvents, which might increase health risk.

Also, the air concentrations of aliphatic organic solvents, including acetone, ethyl acetate and alcohols ethanol, and isopropanol, were lower than their OELs. Among them, ethanol has no OEL at the EU level, yet in the Netherlands, the respective 8 h TWA OEL of ethanol and STEL were set to 260 and 1900 mg/m^3^ due to carcinogenic properties [29].

### 4.5. Other Chemicals

More than 30 chemicals were measured for the first time and in only one study, which illustrates that still very little is known about all types and levels of chemicals to which the hairdressers are exposed to.

There were only two studies that investigated indoor concentrations of persulfates, which are of special concern for hairdressers’ health [37]. Persulfate salts are potent oxidizing agents in hair bleach products that accelerate the bleaching process and are widely used to lighten or bleach hair. Persulfates are the main cause of occupational rhinitis and asthma in hairdressers and one of the leading causes of occupational asthma in some European countries [62,63]. At the EU level, there is no OEL, but several European countries use the OEL proposed by the American Conference for Governmental Industrial Hygienists of 0.1 mg/m^3^ [64]. The average air concentrations of persulfate reported in the study of Mounier-Geyssant et al. [37] amounted to 0.02 mg/m^3^; however, in some salons, the concentrations up to 0.12 mg/m^3^ were measured. In the study of Albin et al. [37], even higher concentrations of persulfates were measured, ranging up to 0.49 mg/m^3^. The fact that such a strong and frequently used sensitizer has been poorly studied indicates that further research and prevention measures are needed.

Another study that should be highlighted is that of Shao et al. [46], investigating for the first time air concentrations of particulate matter (PM). The majority of PM measured in that study included small particles with diameters < 2.5 μm. Smaller particles can penetrate the airway and reach the alveolar region of the lung, thus exacerbating airway and respiratory diseases such as asthma and chronic obstructive pulmonary disease (COPD). Exposure to ambient PM ≤ 2.5 μm in aerodynamic diameter is the most important environmental factor in the global burden of COPD [65]. The study showed that hairdressers in the salons could be potentially overexposed to respirable PM (RPM; defined as particles with a median aerodynamic diameter <4 μm) during an 8 h shift. The median concentration of all the 8 h TWAs for RPM was 299 μg/m^3^, and the highest measured value was 2.1 mg/m^3^. In European countries, the OELs for 8 h TWA exposure to (inert) respirable dust range from 0.3 to 6 mg/m^3^ [26].

## 5. Conclusions

Hairdressers are exposed through inhalation to a wide spectrum of chemicals which may potentially lead to adverse health effects. Airborne concentrations of pollutants depend on salon characteristics such as ventilation and number of customers, but also on used products that are often specific for the country or clientele. This information should be considered when developing and implementing preventive measures. Further, it seems reasonable that information on health risks and protection measures should be provided to hairdressers at an early stage in their career—preferably already in hairdressing schools. Several chemicals that have been detected in the indoor air deserve action. The levels of TVOC, known to be able to exert a wide range of health effects, often exceeded the proposed target or guidance levels for indoor air quality. Combined exposure to TVOC with known endocrine and reproductive effects is of concern as a majority of hairdressers are of reproductive age. In addition, hairdressers are daily exposed to ammonia and persulfates, known to cause respiratory disorders. Although formaldehyde, a human carcinogen, is strictly regulated and severely restricted in the EU market, it is still used in other countries.

These data suggest a need to improve regulations for these chemicals in hairdressing salons. This scoping review reveals that there are numerous compounds that have been investigated in only one or two studies. Thus, more information on the daily exposure of hairdressers to these compounds is needed to perform an adequate risk assessment. A more standardised methodology will aid in comparing findings between studies. Safety assessment for hairdressing products should take into account occupational exposure and not only focus on consumer safety.

## Figures and Tables

**Table 1:** Summary of studies investigating formaldehyde air concentrations (*n* = 8).

1st Author, Year of Publication	Workplace/Country	Number of Workplaces/Total Number of Workers	Exposure Measurement Conditions	Ventilation System	Air Concentrations * Mean ± SD (Range) [μg/m^3^]
**Environmental Sampling**					
Asare-Donkor, 2020	beauty salons/Ghana	60 salons/not reported	environmental samplers located at central area of the salon; sampling time 30 min	windows and ceiling fan	130.53 ± 81.10 (21–434)
Chang, 2018	hairdressing salons/Taiwan	5 salons/42 workers present during sampling of total 60 workers	environmental sampling; sampling time 5 h at a height of 1.3 m above the floor at various areas in the salon	80% of the salons air conditioner, 20% air conditioner plus heat-recovery ventilation	338 (12.4–1040)
Hadei, 2018	beauty salons/Iran	20 salons/184 active cosmetologists	environmental sampling for 3 × 30 min at a height of 1.5 m in the working areas	60% fan, 30% fan plus open window, 10% air purifier	11.88 ± 5.89 (2.94–21.69)
Labreche, 2003	hairdressing salons/Canada	26 salons/not reported	personal and environmental sampling; sampling time from 15 min to 8 h during the busiest days	42% natural ventilation (windows only); 58% general mechanical ventilation	all personal and environmental sampling 40 (20–60)
Peteffi, 2015	beauty salons/Brazil	6 salons/50 workers	environmental samplers placed within the workers’ respiration zone; sampling time 8 h	not reported	median 127.5 ** (IQR 49–172)
Aglan, 2018	hairdressing salons/Egypt	not reported/29 short term employed hairdressers (<5 years)	personal sampling; sampling time 15 min during the second step of the hair straightening procedure	not reported	** 2060.3 ± 331.1
		not reported/31 long term employed hairdressers (>5 years)	environmental sampling; sampling time 15 min during the second step of the hair straightening procedure	not reported	** 2244.3 ± 196.2
Barbosa, 2019	beauty salons/Brazil	10 salons/8 low-exposed hairdressers	personal sampling; sampling time 8 h	not reported	median 15.94 ** (IQR 12.26–24.53)
		10 salons/15 medium-exposed hairdressers			median 42.92 ** (IQR 24.53–73.58)
		10 salons/26 high-exposed hairdressers			median 85.85 ** (IQR 61.32–208.49)
Pexe, 2019	beauty salons/Brazil	23 salons/not reported	personal sampling for 15 min during hair straightening; personal sampling at the height of the personal breathing zone for 8 h	windows and doors opening, reported use of fans in 2 of 23 salons	** 1672.8 ± 1280.3 (0–5083.4) ** 491.9 ± 524.9 (79.7–2275.0)

SD = standard deviation; IQR = interquartile range. * unless otherwise indicated; ** concentrations reported in ppm were calculated in µg/m^3^ as given in the methods section.

**Table 2:** Summary of studies reporting ammonia air concentrations (*n* = 5).

1st Author, Year of Publication	Workplace/Country	Number of Workplaces/Total Number of Workers	Exposure Measurement Conditions	Ventilation System	Air Concentrations * Mean ± SD (Range) [μg/m^3^]
Mendes, 2011	hairdressing salons/Portugal	50 salons/not reported	environmental sampling; sampling time 30 min; samplers placed in areas for storage and handling of the hair dye mixtures and area of hair dye application	62% open windows, 20% mechanical ventilation, 62% air conditioning	** 1598.4 ± 1528.9 (6.95–220,037)
Mounier-Geyssant, 2006	hairdressing salons/France	not reported/53 hairdressing apprentices	environmental sampling; samplers placed in the area where customers sit during and after applying permanent waving, hair colouring or bleaching, near the hair wash area and in the technical room where chemical mixtures are prepared; sampling time 5–8 h	two-thirds of customer spaces and one-third of technical spaces had a ventilation device (fan, air conditioning or other types of venting, e.g., ceiling fan)	680 ± 420 (130–2690)
			personal sampling; sampling time 5–8 h		900 ± 760 (20–4490)
Nemer, 2015	hairdressing salons/Palestine	13 salons/2 to 10 hairdressers per salon	personal sampling; sampling duration from 45 to 305 min	6 of 13 salons had no windows (three of which had air conditioning), two had only holes in the wall allowing for fresh air ventilation, remaining five of 13 salons had one window	12,310 (0–202,100)
Oikawa, 2012	beauty salons/Japan	1 salon/5 hairdressers	environmental sampling; samplers placed close to hair treatment stations during perm treatment; sampling time 1 h, 3–4 times per day	mechanical ventilation	484 ± 243 (150–870)
Ronda, 2009	hairdressing salons/Spain	10 salons/2 to 7 hairdressers per salon	environmental sampling; sampler placed in mixing area; sampling time 3 h, 2 times per day	none of the salons had any general mechanical ventilation or local exhaust ventilation	1800 (400–5100)

SD = standard deviation; IQR = interquartile range. * unless otherwise indicated; ** concentrations reported in ppm were calculated in µg/m^3^ as given in the methods section.

**Table 3:** Summary of studies reporting air concentrations of organic solvents: total volatile organic compounds (TVOC) (*n* = 5), toluene (*n* = 4), xylene (isomers) (*n* = 5), acetone (*n* = 3), benzene (*n* = 3), ethanol (*n* = 3), isopropanol (*n* = 3), ethylacetate (*n* = 3), and ethyl benzene (*n* = 3).

1st Author, Year of Publication	Workplace/Country	Number of Workplaces/Total Number of Workers	Exposure Measurement Conditions	Ventilation System	Air Concentrations * Mean ± SD (Range) [μg/m^3^]
**TVOC**					
De Gennaro, 2014	hairdressing salons/Italy	12 salons/average of 2 hairdressers per salon	environmental sampling; samplers placed in such way to avoid any direct emissions during working tasks; sampling time 24 h (working week days)	Air conditioning	911.1 ± 770.0 (24.24–5002.86)
Mendes, 2011	hairdressing salons/Portugal	50 salons/134 hairdressers	environmental samplers were placed at least 0.6 m above the floor, below the ceiling and away from windows and doors, in rinsing, drying and technical areas; sampling time 45 min	62% open windows, 20% mechanical ventilation, 62% air conditioning	1400 ± 1200 (20–4700)
Moda, 2019	hairdressing salons/UK	5 salons/average of 2 hairdressers per salon	environmental sampling; at 1 m above the floor; sampling time at 15 min intervals during working hours	not reported	** 8909.7 ± 15,580 (949–28,446)
Ronda, 2009	hairdressing salons/Spain	10 salons/not reported, max. 2–7 hairdressers at work in individual salons (28 environmental measurements and 56 personal)	environmental sampling; sampler placed in mixing area; sampling time 3 h, 2 times per day	None of the salons had any general mechanical ventilation or local exhaust ventilation	76,000 (38,000–250,000)
			personal sampling; in the breathing zone of the working hairdressers		107,000 (48,000–237,000)
Ma, 2010	hairdressing salons/Taiwan	62 salons/not reported	personal sampling; sampling time 12 h on working days	Not reported	** 308.0 ± 193.0
**Toluene**					
Hadei, 2018	hairdressing salons/Iran	20 salons/not reported	environmental sampling for 3 × 30 min at a height of 1.5 m in the working areas	60% fan, 30% fan plus open window, 10% air purifier	9.18 ± 3.03 (4.42–14)
Labreche, 2003	hairdressing salons/Canada	26 salons/not reported	personal and environmental sampling; sampling time 15 min to 8 h, during the busiest days	42% natural ventilation (windows only)/58% general mechanical ventilation	270 (20–1660) personal 540 (20–8370) all samples
Moradi, 2019	beauty salons/Iran	36 salons/36 beauty practitioners	environmental sampling for 30 min at a height of 1.5 m in the working areas	not reported	median 98.5 (IQR 70.2–138.9)
Ronda, 2009	hairdressing salons/Spain	10 salons/not reported	personal sampling in the breathing zone of working hairdressers environmental sampling at a height of 1.5 m above the floor in the mixing area; sampling was performed over one week for periods of 3 h	none of the salons had any general mechanical ventilation or local exhaust ventilation	300 (10–3990) personal 120 (20–310) environmental
**m-xylene**					
Moradi, 2019	beauty salons/Iran	36 salons/36 beauty practitioners	environmental sampling for 30 min at a height of 1.5 m in the working areas.		median 16.6 (IQR 12.8–20.8)
Hadei, 2018	hairdressing salons/Iran	20 salons/not reported	environmental sampling for 3 × 30 min at a height of 1.5 m in the working areas	60% fan, 30% fan plus open window, 10% air purifier	11.23 ± 2.57 (7.62–17.23)
Ronda, 2009	hairdressing salons/Spain	10 salons/not reported	personal sampling in the breathing zone of working hairdressers or environmental sampling at a height of 1.5 m above the floor in the mixing area; sampling was performed over one week for periods of 3 h	none of the salons had any general mechanical ventilation or local exhaust ventilation	20 (0–20) personal 20 (0–20) environmental
**p-xylene**					
Ronda, 2009	hairdressing salons/Spain	10 salons/not reported	personal sampling in the breathing zone of working hairdressers or environmental sampling at a height of 1.5 m above the floor in the mixing area. Sampling was performed over one week for periods of 3 h	none of the salons had any general mechanical ventilation or local exhaust ventilation	10 (0–90) personal 10 (0–30) environmental
**o-xylene**					
Moradi, 2019	beauty salons/Iran	36 salons/36 beauty practitioners	environmental samples; sampling for 30 min at a height of 1.5 m in the working areas.	not reported	median 7.7 (IQR 5.6–9.3)
Hadei, 2018	hairdressing salons/Iran	20 salons/not reported	environmental sampling for 3 × 30 min at a height of 1.5 m in the working areas	60% fan, 30% fan plus open window, 10% air purifier	6.78 ± 1.92 (3.12–9.44)
Ronda, 2009	hairdressing salons/Spain	10 salons/not reported	personal sampling in the breathing zone of working hairdressers or environmental sampling at a height of 1.5 m above the floor in the mixing area; sampling was performed over one week for periods of 3 h	none of the salons had any general mechanical ventilation or local exhaust ventilation	10 (0–60) personal 10 (0–30) environmental
**Benzene**					
Hadei, 2018	hairdressing salons/Iran	20 salons/not reported	environmental sampling for 3 × 30 min at a height of 1.5 m in the working areas	60% fan, 30% fan plus open window, 10% air purifier	7.54 ± 1.87 (4.73–11.33)
Moradi, 2019	beauty salons/Iran	36 salons/36 beauty practitioners	environmental sampling for 30 min at a height of 1.5 m in the working areas.	not reported	median 4.3 (IQR 3.4–5.7)
Ronda, 2009	hairdressing salons/Spain	10 salons/not reported	personal sampling in the breathing zone of working hairdressers or environmental sampling at a height of 1.5 m above the floor in the mixing area; sampling was performed over one week for periods of 3 h	none of the salons had any general mechanical ventilation or local exhaust ventilation	10 (0–20) personal 10 (0–20) environmental
**Ethyl benzene**					
Hadei, 2018	hairdressing salons/Iran	20 salons/not reported	environmental sampling for 3 × 30 min at a height of 1.5 m in the working areas	60% fan, 30% fan plus open window, 10% air purifier	14.00 ± 4.21 (7.68–22.10)
Moradi, 2019	beauty salons/Iran	36 salons/36 beauty practitioners	environmental sampling for 30 min at a height of 1.5 m in the working areas.	not reported	median 6.8 (IQR 5.6–8.3)
Ronda, 2009	hairdressing salons/Spain	10 salons/not reported	personal sampling in the breathing zone of working hairdressers or environmental sampling at a height of 1.5 m above the floor in the mixing area; sampling was performed over one week for periods of 3 h	none of the salons had any general mechanical ventilation or local exhaust ventilation	10 (0–50) personal 10 (0–40) environmental
**Ethanol**					
Chang, 2018	hairdressing salons/Taiwan	5 salons/not reported	environmental sampling with a sampling time of 5 h at a height of 1.3 m above the floor at various areas in the salon	80% of the salons air conditioner, 20% air conditioner plus heat-recovery ventilation	1860 (157–10,500)
Labreche, 2003	hairdressing salons/Canada	26 salons/not reported	personal and environmental; sampling time 15 min to 8 h during the busiest days	42% natural ventilation (windows only)/58% general mechanical ventilation	39,900 (170–447,170) personal 46,300 (170–447,170) all samples
Ronda, 2009	hairdressing salons/Spain	10 salons/not reported	personal sampling in the breathing zone of working hairdressers or environmental sampling at a height of 1.5 m above the floor in the mixing area; sampling was performed over one week for periods of 3 h	None of the salons had any general mechanical ventilation or local exhaust ventilation	2950 (0–43,120) personal 2160 (0–6360) environmental
**Iso-propanol**					
Chang, 2018	hairdressing salons/Taiwan	5 salons/not reported	environmental sampling, with a sampling time of 5 h at a height of 1.3 m above the floor at various areas in the salon	80% of the salons air conditioner, 20% air conditioner plus heat-recovery ventilation	208 (14.5–1240)
Labreche, 2003	hairdressing salons/Canada	26 salons/not reported	personal and environmental; sampling time 15 min to 8 h during the busiest days	42% natural ventilation (windows only)/58% general mechanical ventilation	3130 (40–28,870) personal 3280 (40–28,870) all samples
Ronda, 2009	hairdressing salons/Spain	10 salons/not reported	personal sampling in the breathing zone of working hairdressers or environmental sampling at a height of 1.5 m above the floor in the mixing area; sampling was performed over one week for periods of 3 h	none of the salons had any general mechanical ventilation or local exhaust ventilation	600 (10–2970) personal 780 (10–2900) environmental
**Acetone**					
Chang, 2018	hairdressing salons/Taiwan	5 salons/not reported	environmental sampling, with a sampling time of 5 h at a height of 1.3 m above the floor at various areas in the salon	80% of the salons air conditioner, 20% air conditioner plus heat-recovery ventilation	77.9 (1.86–260)
Labreche, 2003	hairdressing salons/Canada	26 salons/not reported	personal and environmental; sampling time from 15 min to 8 h during the busiest days	42% natural ventilation (windows only)/58% general mechanical ventilation	3770 (810–22,240) personal 6360 (810–22,450) all samples
Ronda, 2009	hairdressing salons/Spain	10 salons/not reported	personal sampling in the breathing zone of working hairdressers or environmental sampling at a height of 1.5 m above the floor in the mixing area Sampling was performed over one week for periods of 3 h	none of the salons had any general mechanical ventilation or local exhaust ventilation	310 (10–2320) personal 400 (10–2420) environmental
**Ethyl acetate**					
Chang, 2018	hairdressing salons/Taiwan	5 salons/not reported	environmental sampling, with a sampling time of 5 h at a height of 1.3 m above the floor at various areas in the salon	80% of the salons air conditioner, 20% air conditioner plus heat-recovery ventilation	190 (0.3–1310)
Labreche, 2003	hairdressing salons/Canada	26 salons/not reported	personal and environmental; sampling time from 15 min to 8 h, during the busiest days	42% natural ventilation (windows only)/58% general mechanical ventilation	1340 (180–3440) personal 1370 (130–4470) all samples
Ronda, 2009	hairdressing salons/Spain	10 salons/not reported	personal sampling in the breathing zone of working hairdressers or environmental sampling at a height of 1.5 m above the floor in the mixing area; sampling was performed over one week for periods of 3 h	none of the salons had any general mechanical ventilation or local exhaust ventilation	1190 (0–14,740) personal 1430 (0–14,820) environmental

* unless otherwise indicated; ** concentrations reported in ppm were calculated in µg/m^3^ as given in the methods section. SD = standard deviation; IQR = interquartile range.

## Data Availability

All data and references are included in the manuscript.

## Supplementary Materials

The following supporting information can be downloaded at: https://www.mdpi.com/article/10.3390/ijerph19074176/s1, Table S1. Search strategies for MEDLINE and EMBASE; Table S2. Occupational exposure limits; Table S3. Summary of studies investigating air concentration of individual chemicals reported in one or two studies.

## Abbreviations

OEL	Occupational exposure limit
PEL	Permissible exposure limits
STEL	Short time exposure limit
TWA	Time-weighted average
TVOC	Total volatile organic compounds
