# Supplementary material for: Occupational Exposure of Hairdressers to Airborne Hazardous Chemicals: A Scoping Review
Source: Int J Environ Res Public Health. 2022 Mar 31;19(7):4176. doi: 10.3390/ijerph19074176 (PMC8998463; doi:10.3390/ijerph19074176)
Supplement: Supplementary file 1 [file ijerph-19-04176-s001.zip › ijerph-1598740-supplementary.pdf]

Table S1. Search strategies for MEDLINE and EMBASE.

	**Ovid MEDLINE(R) ALL <1946 to March 31, 2021> Search date: 1 April 2021**	
#	**Searches**	**Results**
1	barbering/ or hair preparations/	1769
2	(hairdres* or barber* or hair dres* or beautician apprentice* or (apprentice* and hair)).ab,kf,ti.	2884
3	1 or 2 [hairdressers]	4208
4	(Hair adj3 (wash* or shampoo* or dye? or dying or lotion* or co?loring or bleach* or perm or perming or curl* or styling or condition* or straightening)).ab,kf,ti.	3276
5	wet work.ab,kf,ti.	195
6	4 or 5 [specific occupational exposure]	3464
7	air pollutants, occupational/ or volatile organic compounds/ or air pollution, indoor/ or ammonia/	65742
8	(air pollut* or volatile organic compound? or ammonia).ab,kf,ti.	94697
9	("106-50-3" or "624-18-0" or "16245-77-5" or "95-70-5" or "615-50-9" or "337906-36-2" or "337906-37-3" or "7727-54-0" or "7727-21-1" or "7775-27-1" or "30618-84-9" or "5421-46-5" or "868-77-9" or "7085-85-0").ab,kf.	7
10	or/7-9 [generic occupational exposure]	134468
11	exp employment/ or exp work/ or "rehabilitation, vocational"/ or occupational health/ or occupational groups/ or sick leave/ or workers' compensation/	188269
12	(paid work or worker? or vocational or occupation* or sick leave or absenteeism or presenteeism or employment or employee? or insurance medicine or sickness absence or job? or work abil* or work participa* or work place or "return to work" or work related or work disabil* or work product* or work limit* or work instabili* or work function* or work performance or work capacit or work evaluat* or work direct* or working populat* or workplace or disability benefi* or sickness benefi* or legal compensation or disability compensation or disability benefit? or sickness benefit?).ab,kf,ti.	492107
13	(compensation adj2 (claim* or income)).ab,kf,ti.	1731
14	(receiv* adj2 compensation).ab,kf,ti.	702
15	or/11-14 [work-related]	595102
16	(3 or 6) and 15	1085
17	(3 or 6) and 10	99
18	16 or 17	1142
19	exp animals/ not humans/	4807263
20	18 not 19	1131
21	limit 20 to yr="2000-current"	809
22	remove duplicates from 21	806
	**Ovid Embase Classic+Embase <1947 to 2021 March 31> Search date: 1 April 2021**	
#	**Searches**	**Results**
1	hairdresser/ or (cosmetic/ and hair.mp.)	4005
2	(hairdres* or barber* or hair dres* or beautician apprentice* or (apprentice* and hair)).ab,kw,ti.	4191

3	1 or 2 [hairdressers]	6508
4	hair bleaching agent/ or hair color/ or hair dye/	5398
5	(Hair adj3 (wash* or shampoo* or dye? or dying or lotion* or co?loring or bleach* or perm or perming or curl* or styling or condition* or straightening)).ab,kw,ti.	4765
6	wet work.ab,kw,ti.	352
7	or/4-6 [specific occupational exposure]	8808
8	exp air pollution/ or volatile organic compound/ or ammonia/	256508
9	(air pollut* or volatile organic compound? or ammonia).ab,kw,ti.	124836
10	("106-50-3" or "624-18-0" or "16245-77-5" or "95-70-5" or "615-50-9" or "337906-36-2" or "337906-37-3" or "7727-54-0" or "7727-21-1" or "7775-27-1" or "30618-84-9" or "5421-46-5" or "868-77-9" or "7085-85-0").ab,kw,rn.	6558
11	or/8-10 [more generic occupational exposure]	296770
12	exp employment/ or work/ or absenteeism/ or presenteeism/ or return to work/ or work capacity/ or workplace/ or vocational rehabilitation/ or occupational health/ or "named groups by occupation"/ or medical leave/ or workman compensation/	266404
13	(paid work or worker? or vocational or occupation* or sick leave or absenteeism or presenteeism or employment or employee? or insurance medicine or sickness absence or job? or work abil* or work participa* or work place or "return to work" or work related or work disabil* or work product* or work limit* or work instabili* or work function* or work performance or work capacit or work evaluat* or work direct* or working populat* or workplace or disability benefi* or sickness benefi* or legal compensation or disability compensation or disability benefit? or sickness benefit?).ab,kw,ti.	654560
14	(compensation adj2 (claim* or income)).ab,kw,ti.	2207
15	(receiv* adj2 compensation).ab,kw,ti.	998
16	or/12-15 [work-related]	764712
17	(3 or 7) and 16	1976
18	(3 or 7) and 11	907
19	17 or 18	2661
20	(animal/ or animal experiment/ or animal model/ or nonhuman/ or rat/ or mouse/ or (rat or rats or mouse or mice).ti.) not human/	7158857
21	19 not 20	2561
22	limit 21 to yr="2000-current"	1909
23	remove duplicates from 22	1882

Table S2. Occupational exposure limit values in the EU and OSHA

	**EU[1]**		OSHA[2]	
	**8 hr TWA**	15 min TWA	8 hr-TWA	15 min TWA
	mg/m^3	mg/m^3	mg/m^3	mg/m^3
Acetone	1210	na	1000	2400
Ammonia	14	36	35	na
Benzene	3.25	na	3.2	15.6
Ethanol	na	na	1000	1900
Ethyl acetate	734	1468	1400	na
Formaldehyde	0.37	0.74	0.92	2.5
iso-propanol	na	na	980	na
Toluene	192	384	754	1130
xylene	221	442	435	na

TWA: Time weighted average

[1] Insurance, I. F. O. S. a. H. o. t. G. S. A. GESTIS - International limit values for chemical agents (Occupational exposure limits, OELs).

https://www.dguv.de/ifa/gestis/gestis-internationale-grenzwerte-fuer-chemische-substanzen-limit-values-for-chemical-agents/index-2.jsp,

[2] (OSHA), O. S. H. A. Permissible Exposure Limits. https://www.osha.gov/annotated-pels.

Table S3. Summary of studies investigating air concentration of individual chemicals reported in one or two studies.

1st Author, year of publication, country	Workplace, population	Chemicals	Exposure measurement conditions	Ventilation system	Concentration in µg/m3 (range)
Albin 2002, Sweden	1 Hairdressing salon / 3 personal samples	persulphates	Environmental measurement: Stationary sampling with 4 samplers in the mixing area, sampling time: 200–319 minute	Not reported	<4 - 6.1
			Personal sampling: during mixing of bleaching powder with peroxide and application of the mixture, sampling time 32–135 minutes		15 - 490
Chang, 2018,Taiwan	5 hairdressing salons, not reported	butyl benzyl phthalate	sampling time of 5 hours at a height of 1,3 meters above the floor at various areas in the salon.	80% of the salons air conditioner, 20% air conditioner plus heat-recovery ventilation	0.088 (0.005 - 1.91)
		di[2-ethylhexyl]phthalate			1.01 (0.015 – 6.09)
		dimethyl phthalate			2.01 (0.005 - 17.5)
		isobutyl acetate			9 (0.2 - 53.3)
		N-butyl acetate			15.6 (0.2 - 49)
		sec-butyl acetate			4.5 (0.4 - 30.6)
		tert-butyl acetate			1.9 (0.3 - 12.4)
		dibutyl phthalate			0.73 (0.24 - 1.54)
		diethyl phthalate			1.19 (0.15 - 3.57)
Hadei, 2018; Iran	20 hairdressing salons, not reported	acetaldehyde	Environmental sampling for 3x30 minutes at a height of 1,5 meters in the working areas	60% fan, 30% fan plus open window, 10% air purifier	32.67 ± 12.59 (11.79 - 55.93)
Labreche, 2003, France	26 hairdressing salons, not reported	amino-2-ethanol	Personal and environmental. Sampling time ranged from 15 min to 8 hours depending on activities performed by workers, during the busiest days.	42% natural ventilation (windows only)/ 58% general mechanical ventilation	2 (1 - 5) personal<br>2 (1 - 5) all samples (including personal)
		methyl ethyl ketone			100 (70 - 140) personal<br>120 (70 - 180) all samples (including personal)
		butyl acetate			230 (70 - 1000) personal<br>350 (50 - 1510) all samples
		dibutyl phthalate			3 (2 - 3) all samples
Liu, 2018, USA	36 barbershops, not reported	methyl siloxanes D4, D5, D6	In indoor dust	Not reported	3.126 (5293 – 1.326·10^5) ng/g

Liu, 2013, USA	55 barbershops, not reported	Synthetic musks	In indoor dust	Not reported	$7.68 \cdot 10^4$ (790 – $1.2 \cdot 10^6$) ng/g
Mounier-Geyssant, 2006	Not reported / 53 hairdressing apprentices	hydrogen peroxide	Environmental sampling: samplers placed in the technical space of the customer area (where customers sit during and after applying permanent waving, hair colour or bleaches), near the hair wash area and in the 'technical room' (i.e. where chemical mixtures are prepared); sampling time: 5-8 hours	Two thirds of customer spaces and one third of technical spaces had ventilation device (fan, air conditioning or other type of venting, like ceiling fan)	40 ± 30 (3 - 150)
			Personal sampling: sampling time: 5-8 hours		50 ± 40 (3 - 180)
		persulfates	Environmental sampling: samplers placed in the technical space of the customer area (where customers sit during and after applying permanent waving, hair colour or bleaches), near the hair wash area and in the 'technical room' (i.e. where chemical mixtures are prepared); sampling time: 5-8 hours		20 ± 20 (2 - 80)
Oikawa, 2012, Japan	1 beauty salon, 5 hairdressers	dithioglycolic acid	Samples collected in water using an impinge, devices were move closer to hair treatment stations while a perm treatment was performed	Mechanical ventilation, use of a room air cleaner	nc (0 - 750)
		thioglycolic acid			nc (0 - 8)
Ronda, 2009, Spain	10 hairdressing salons, not reported	1-dodecanol	Personal sampling in the breathing zone of working hairdressers or environmental sampling at a height of 1,5 meters above the floor in the mixing area. Sampling was performed over one week for periods of 3 hours	None of the salons had any general mechanical ventilation or local exhaust ventilation	30 (10 - 190) personal<br>10 (0 - 20) environmental
		1-methoxypropan-2-ol			70 (0 - 1390) personal<br>20 (0 - 280) environmental
		2-phenylethanol			10 (0 - 80) personal<br>10 (0 - 50) environmental
		2-propanol, 1-butoxy			210 (0 - 8810) personal<br>480 (0 - 830) environmental

	a-isomethyl ionone	20 (0 - 260) personal<br>10 (0 - 20) environmental
	benzene, 1,2,4-trimethyl-	10 (0 - 10) personal<br>10 (0 - 10) environmental
	butane	3420 (20 - 52520) personal<br>2850 (60 - 15470) environmental
	butane, 2-methyl	2620 (0 - 39260) personal<br>3820 (0 - 37710) environmental
	butanol	0.03 (0.00 - 0.02) personal<br>10 (0 - 60) environmental
	dekametylsylopentasiloksan	14760 (90 - 211470) personal<br>7670 (100 - 29460) environmental
	dichloromethane	250 (0 - 1830) personal<br>120 (0 - 700) environmental
	dimethylether	50 (0 - 90) personal<br>0 (0 - 0) environmental
	dimethylbenzene-ol	190 (20 - 2230) personal<br>150 (10 - 1120) environmental
	diphenyl ether	20 (10 - 130) personal<br>10 (0 - 20) environmental
	heptamethylnonane	80 (0 - 1320) personal<br>60 (0 - 1160) environmental
	hexane	10 (0 - 130) personal<br>10 (0 - 50) environmental
	isobornyl acetate	20 (0 - 250) personal<br>10 (0 - 30) environmental
	limonene	1280 (20 - 17690) personal<br>1440 (30 - 10610) environmental
	propylene glycol	30 (10 - 130) personal<br>20 (0 - 90) environmental
	siloksane	1670 (0 - 22260) personal<br>1320 (0 - 12280) environmental

		butyl acetate			386 (0 - 89660) personal<br>4540 (0 - 105430) environmental
		diethyl phthalate			480 (10 - 10970) personal<br>120 (0 - 750) environmental
Shao, 2021	3 Salons primarily serving African/African American clientele, not reported	particulate matter 2,5	Air sampling for 8 hours per workday at a height of 1,5 meters above the floor	Not reported	221.2 ± 125.8 (16 – 380)
	3 Salons primarily serving Dominican clientele, not reported				734.3 ± 606.6 (8 – 2068)
Wan, 2015	5 barbershops/hair salons, not reported	benzophenone-3	Air samples were collected for 3 to 24 hours at the place in question	Indirectly measured from CO_2 values. Well ventilated	0.00675 (0.00290 – 0.0194) (geometric mean)
Wan, 2016	Not reported, not reported	benzothiazoles		Indirectly measured from CO_2 values. Well ventilated	0.0189 (0.0101 – 0.0329) (geometric mean)
